# Influence of Fragrances on Human Psychophysiological Activity: With Special Reference to Human Electroencephalographic Response

**DOI:** 10.3390/scipharm84040724

**Published:** 2016-11-29

**Authors:** Kandhasamy Sowndhararajan, Songmun Kim

**Affiliations:** School of Natural Resources and Environmental Sciences, Kangwon National University, Chuncheon 24341, Korea; sowndhar1982@gmail.com

**Keywords:** aroma, brain wave, electroencephalography, fragrance, psychophysiology

## Abstract

The influence of fragrances such as perfumes and room fresheners on the psychophysiological activities of humans has been known for a long time, and its significance is gradually increasing in the medicinal and cosmetic industries. A fragrance consists of volatile chemicals with a molecular weight of less than 300 Da that humans perceive through the olfactory system. In humans, about 300 active olfactory receptor genes are devoted to detecting thousands of different fragrance molecules through a large family of olfactory receptors of a diverse protein sequence. The sense of smell plays an important role in the physiological effects of mood, stress, and working capacity. Electrophysiological studies have revealed that various fragrances affected spontaneous brain activities and cognitive functions, which are measured by an electroencephalograph (EEG). The EEG is a good temporal measure of responses in the central nervous system and it provides information about the physiological state of the brain both in health and disease. The EEG power spectrum is classified into different frequency bands such as delta (0.5–4 Hz), theta (4–8 Hz), alpha (8–13 Hz), beta (13–30 Hz) and gamma (30–50 Hz), and each band is correlated with different features of brain states. A quantitative EEG uses computer software to provide the topographic mapping of the brain activity in frontal, temporal, parietal and occipital brain regions. It is well known that decreases of alpha and beta activities and increases of delta and theta activities are associated with brain pathology and general cognitive decline. In the last few decades, many scientific studies were conducted to investigate the effect of inhalation of aroma on human brain functions. The studies have suggested a significant role for olfactory stimulation in the alteration of cognition, mood, and social behavior. This review aims to evaluate the available literature regarding the influence of fragrances on the psychophysiological activities of humans with special reference to EEG changes.

## 1. Introduction

The aroma components from natural products have been used for mental, spiritual and physical healing since the beginning of recorded history. In aromatherapy, fragrance substances (aroma/odor/scent) from various natural sources have been used for the treatment of various disorders. The aromatherapy treatment is a natural way of healing a person’s mind, body and soul. Many ancient civilizations, including Egypt, China and India, have used aromatherapy as a popular complementary and alternative therapy for more than thousands of years [1]. In traditional medicine as well as in aromatherapy and herbal medicine, essential oils and fragrance compounds have been used for the treatments of various psychological and physical disorders such as headaches, pain, insomnia, eczema, stress-induced anxiety, depression and digestive problems [2,3]. In recent years, various studies have revealed that olfactory stimulation through fragrance inhalation exerts various psychophysiological effects on human beings. There are various methods available to administer the fragrances in small quantities, including inhalation, massage or simple applications on the skin surface and, sometimes, they can be taken internally [4,5].

In our daily life, several fragrances appear and a sense of smell plays an important role in the physiological effects of mood, stress, and working capacity. Fragrance is a volatile chemical component with a molecular weight of <300 Da that humans perceive via the olfactory system. In the olfactory process, the fragrant molecules in the air attach to the cilia of olfactory receptors in the olfactory epithelium, located in the nasal cavity. Then the guanine nucleotide binding protein (G-protein) coupled receptors (GPCR) are activated and electrical signals are generated. Subsequently, the electrical signals are transmitted to the brain by olfactory sensory neurons via olfactory bulb and higher olfactory cortex [5,6]. Consequently, these electrical signals modulate the brain functions including memory, thoughts, and emotions. Many studies describe that the inhalation of fragrances highly affect the brain function since the fragrance compounds are able to cross the blood-brain barrier and interact with receptors in the central nervous system [7,8]. Furthermore, many studies have suggested that the olfactory stimulation of fragrances produces immediate changes in physiological parameters such as blood pressure, muscle tension, pupil dilation, skin temperature, pulse rate and brain activity [5,9,10]. Hence, the studies in relation to the role of fragrances in the brain functions of healthy and diseased subjects have significantly increased in the past decades.

There are numerous techniques that have been developed to examine the brain function. The emotional and behavior alterations by fragrance inhalation have been assessed by different electrophysiological methods such as electroencephalograph (EEG), contingent negative variation, near infrared spectroscopy, and functional magnetic resonance imaging [5,11,12]. Among them, EEG is the best temporal measure of responses in the central nervous system and is susceptible to alteration during exposure to fragrance. Furthermore, the perfect classification of electrical activity for a particular state of human brain supports the diagnoses of neurological diseases. Previous studies reported that the odors affected spontaneous brain activities and cognitive functions were estimated by EEG [13,14,15,16,17]. The EEG power spectra were estimated by using Fast Fourier Transform that allows the quantitative analysis of electrical signals in the total as well as in single frequency bands. The EEG spectrum is a complex signal resulting from postsynaptic potentials of cortical pyramidal cells and these signals can be recorded by the metal electrodes placed on the surface of scalp [18]. Based on the above knowledge, we present an overview of scientific experimentation in regards to psychophysiological effects of fragrances with special reference to EEG studies.

## 2. Fragrance Components

The fragrances are mainly volatile organic compounds with characteristic, usually pleasant odors. They have been used for thousands of years to deliver a variety of benefits, especially for the physical and psychological well-being of humans. In the 1920s, Rene-Maurice Gattefosse, a French chemist, coined the term aromatherapy, referring specifically to the use of natural fragrance essential oils to treat injury and disease [4]. Nowadays, a variety of consumer products such as candles, perfumes and other personal care products, room fresheners, detergents, etc., are commercially available with aromatherapy benefits. The aromatic properties of these products play a major role in the psychophysiological functions of human beings [1]. The fragrance materials are exposed to consumers ranging from skin contact to inhalation. The fragrance components have some specific molecular properties in order to provide sensory properties. It has a sufficiently high vapor pressure, low polarity, some ability to dissolve in fat and surface activity. Fragrance materials vary from highly complex mixtures to single chemicals. The fragrance molecules are mainly limited to the molecular weight of 200 to 300 Da but within that range, there are essentially a vast number of fragrant components and their molecular structures are highly varied. The natural fragrance materials are mainly obtained from plants, resins, animal secretion and their metabolites [19].

Among the various natural fragrant components, essential oils are the main therapeutic agents, which are said to be a highly concentrated volatile and complex mixture of aromatic components obtained from different organs of the plant. There are about 17,500 aromatic plant species from different angiospermic families producing essential oils, particularly Lamiaceae, Rutaceae, Myrtaceae, Zingiberaceae and Asteraceae. The essential oils contain approximately 20–60 different components at various concentrations. They are characterized by two or three major components at relatively higher levels (20%–70%) with several other minor components (trace amounts) [20,21]. In general, these major components are responsible for the biological potentials of the essential oils. The components of essential oils are classified into two major groups (terpenes and aromatic compounds) based on their biosynthetic origin. The terpenes are the largest group of natural fragrances. The classification of terpenes is mainly based on the number of isoprene units present in their structure. Depending on the number of C_5_ units, the terpenes are classified into hemiterpenes (C_5_), monoterpenes (C_10_), sesquiterpenes (C_15_) and diterpenes (C_20_). Based on the functional groups, the terpenes and other aromatic compounds have been classified into hydrocarbons, alcohols, aldehydes, ketones, phenols, esters, ethers, etc. [19,22,23]. Thousands of different terpene and aromatic structures occur in perfume ingredients, both natural and synthetic.

In the terpenes, monoterpenes are the most abundant molecules of the essential oils (about 90%) with a great variety of structures. Geraniol/nerol, linalool, citronellol, citronellal and citral are the most important terpenes and are widely used in the perfume industries [6,21]. In aromatherapy, the medicinal and aromatic plants including bergamot, caraway, eucalyptus, geranium, juniper, lavender, lemon, lemongrass, mint, orange, peppermint, pine, rosemary, sage, tea tree, thyme and ylang-ylang have been used to cure a variety of physical and psychological disorders. These plants contain various bioactive monoterpene and sesquiterpene components along with other aromatic components. Table 1 shows the names of some important essential oil-bearing plants with their major bioactive components [1,21,22]. Some of the fragrance components from animal origin such as macrocyclic ketones and esters as well as aromatic nitro compounds and polycyclic aromatics (group of musk fragrances) are also widely used in the perfume industries. The natural fragrances from plants and animals were predominantly used until the end of the 19th century. At present, synthetic fragrances are increasingly applied due to the constant and reproducible quality over natural fragrances [24].

## 3. The Olfactory Process

Olfaction is a prehistoric sense for humans and animals. It permits vertebrates and other organisms with an olfactory system to identify food, mates, predators, and provides both sensual pleasure as well as warnings of danger, such as spoiled food or chemical hazards. In humans and animals, it is one of the important means by which our environment communicates with us. Previous studies stated that even a small amount of fragrance compounds taken by respiration causes indirect physical effect by activating olfactory memory. In addition, the fragrance and the sense of smell are very important in the direction of human behavior [7,8].

**Figure 1:**
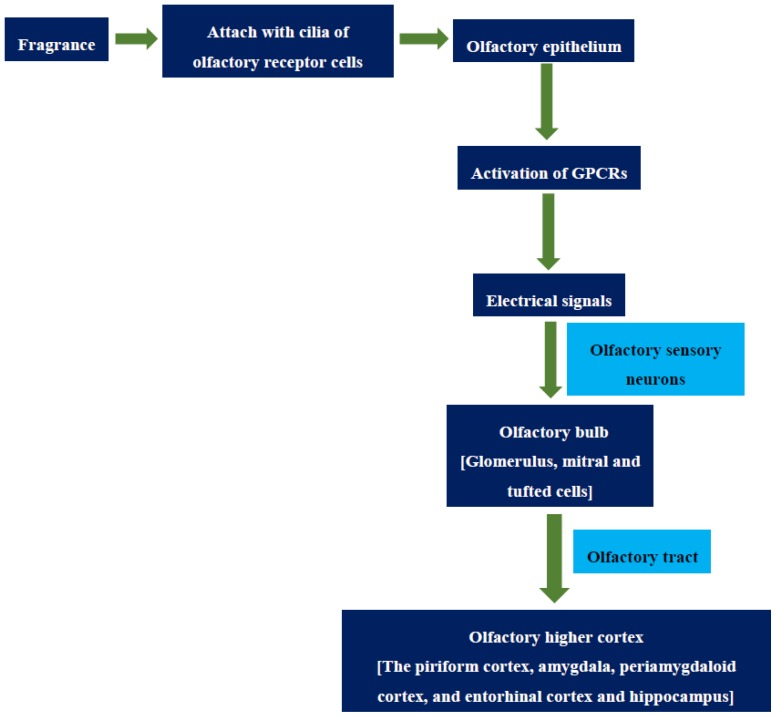
Schematic representation of the olfactory process. GPCR: guanine nucleotide binding protein coupled receptors.

The olfactory system contains a sensory organ (olfactory epithelium) and specific olfactory brain regions (olfactory bulb and higher olfactory cortex). The olfactory mucosa is the region which is located in the superior and posterior part of the nasal cavity, especially for the detection of fragrant molecules. It includes the olfactory epithelium and its underlying lamina propria. In general, the olfactory epithelium undergoes a continual process of neurogenesis in which new neurons are constantly generated throughout adult life, and this is the reason for discontinuity and spread of the olfactory mucosa [25]. The olfactory receptor cells contain cilia where molecular reception with the fragrance occurs and sensory transduction starts [26]. At one side, the olfactory receptor neurons extend through the epithelium to contact fragrant molecules in the air. At the other end, the olfactory receptor cells in the epithelium form axons to penetrate the cribriform plate of bone, reaching the olfactory bulb of the brain where they converge to terminate with post-synaptic cells to form synaptic structures called glomeruli. Each glomerulus receives input from olfactory sensory neurons expressing the same type of odorant receptor. The glomeruli are connected in groups that converge into mitral cells and tufted cells. The mitral cells and tufted cells are the primary efferent projection neurons of the olfactory bulb. From the mitral cells, the olfactory information is transmitted directly to the higher olfactory cortex in the corticomedial amygdala portion of the brain through olfactory tract where the signaling process is decoded and olfactory interpretation and response occurs (Figure 1) [27,28,29]. 

The olfactory process begins when fragrances from the atmosphere enter into the nose and attach to the cilia of receptor cells. In general, fragrance stimulation of olfactory receptor cells in the nose involves interaction of fragrance molecules with olfactory-receptor proteins. Buck and Axel [30] found the family of transmembrane proteins believed to be the odor receptors and some of the genes that encode them. They found that the proteins contained the seven-helical transmembrane structure and contained sequence resemblance to other members of the G-protein-coupled receptor family. The previous studies reported that humans have about 350 active odorant receptor genes and about 560 odorant receptor pseudogenes. The olfactory events undergo two different G-protein-coupled transduction mechanisms; one activating adenylyl cyclase to generate cyclic adenosine monophosphate, the other activating phospholipase C to produce inositol trisphosphate. Then they open channels admitting calcium, sodium and chlorine ions into the cell, leading to depolarization of the membrane and an action potential. Then the olfactory sensory neurons send the electrical signals to the brain via olfactory bulb and higher olfactory cortex [6,27,29]. The discovery of the olfactory receptor genes provides new genetic and molecular techniques for pursuing the organization of the olfactory pathway in the brain.

The olfactory system is described by relatively direct connections to brain structures involved in memory and emotion such as the hippocampus, thalamus, and frontal cortex. The olfactory tract carries the axons leaving the olfactory bulb and projects to the structures collectively called the primary olfactory cortex [26]. The piriform cortex is the largest of the olfactory areas and occupies a central position in the primary olfactory cortex. The piriform cortex in humans appears to be involved in odor recognition memory. The other important cortical primary olfactory areas include the anterior cortical nucleus of the amygdala, the periamygdaloid cortex, and the entorhinal cortex [31,32]. Olfactory information is transmitted from the primary olfactory cortex to other cortical and subcortical areas. Further, the periamygdaloid and the entorhinal cortex provide olfactory information to the amygdala and hippocampus [28,29].

## 4. Electroencephalography (EEG)

An EEG is a recording of fluctuating electrical waveforms at the scalp of human brain. Diagnostic applications of EEG include epilepsy, disturbances of consciousness, brain death, dementia, cerebrovascular or structural brain disease, and other psychological disorders. The practical application of EEG holds great promise for increasing our understanding of human central nervous system activity in relation to the influence of fragrances on brain function [33,34]. In recent years, a number of advanced techniques have become available to study the influence of fragrances on brain function. The psychophysical measures and brain imaging techniques are widely used to detect brain activity. Previous studies have reported that the EEG studies are effectively used to understand spontaneous brain activities and cognitive functions through fragrance inhalation [16,17,35]. Further, those studies clearly suggested that human EEG activity is susceptible to alteration during exposure to fragrance.

The EEG power spectra were determined by Fast Fourier Transform that allows the quantitative analysis of signals in the total as well as in single frequency bands. The neuronal activity in the brain was detected by recording the EEG signals from the scalp or the surface of the brain. The EEG signals arises from excitatory (depolarizing) and inhibitory (hyperpolarizing) post-synaptic potentials in populations of pyramidal neurons, which are located in the lower layers of the cerebral cortex [33,36]. The EEG power spectra bands frequently applied for examining the brain activity are the delta (0–4 Hz), theta (4–8 Hz), alpha (8–13 Hz) beta (13–30 Hz) and gamma (>30 Hz) waves [37].

The EEG recording is relatively simple, non-invasive and could serve as an objective method for evaluating the olfactory system. It is possible to achieve results in a short period of time, and it does not require active cooperation of subjects [16]. Small disc-shaped metal electrodes are fixed to different locations on the subject’s scalp according to the International 10–20 system. Further, electrode gel is used to enhance the contact between the scalp and electrodes. The electrode epidermal impedance must be less than 5 kΩ before reliable recordings can be made. The electrodes detect the sum of positive and negative charges in their vicinity [36]. The studies of odor-related EEG may lead to understanding the psychophysiological activities induced by various fragrances.

### 4.1. Brain Waves and Their Functions

**Figure 2:**
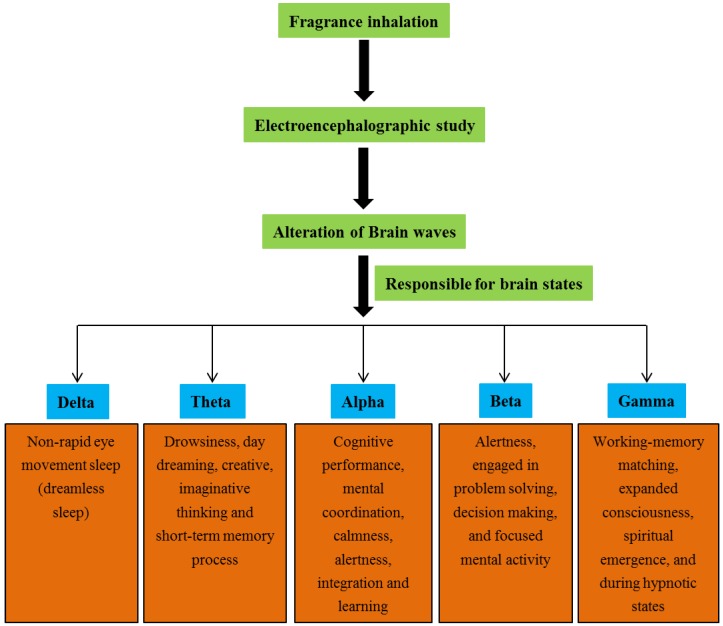
Brain waves and their functions.

Brain waves naturally appear during both the active and resting states. Our thoughts, emotions and behavior are the reflection of neuronal activity within the brain. The activation of brain waves characterizes the neuronal electrical activity, particularly the voltage fluctuations from ionic flows of neurons in the brain. The EEG measures these electrical activities and represents them as waves or oscillations. Commonly, these brain waves are representative of specific functions throughout the brain. The EEG may be affected by sleep, brain disorders, medication and age [37]. There are many separate wave bands ranging from 0.05 to 500 Hz that have been operationally expressed based on different states of the brain function. The brain waves with slower frequencies are dominant when we feel tired, slow, or dreamy. On the other hand, the higher frequencies are dominant when we feel wired or hyper-alert [38]. The following categories of frequency bands are the most therapeutically relevant (Figure 2).

#### 4.1.1. Delta Waves

Delta waves (0–4 Hz) are the slowest and the most important EEG feature of human non-rapid eye movement sleep (dreamless sleep), which have their origin in cortical layers [39]. This kind of sleep is also called slow wave sleep because the EEG activity producing slow waves with a frequency of <1 Hz. They are normal in sleeping adults and children, but abnormal in awakening adults. These waves are the most common focal pathological waveform [40].

#### 4.1.2. Theta Waves

Theta waves occur between the frequencies of 4–8 Hz during sleep and are also dominant in deep meditation. These waves are transiently found in 15% of the normal population and occur in both cortical and hippocampal regions. When compared to adults, children tend to have a significantly higher level of theta activity. The theta state is also connected with subconscious fears, worries and nightmares. Further, these waves indicate sleep, drowsiness, daydreaming, as well as creative and imaginative thinking that is controlled by the subconscious mind. The theta waves play a major role in the function of short-term memory and the process of building memories [37,41,42].

#### 4.1.3. Alpha Waves

Alpha waves have frequencies from 8 to 13 Hz. They occur in all age groups with closed eyes but are prominent in adults. In particular, alpha waves occur while an individual is temporarily idle, but still alert. These waves occur during moderate levels of brain activity and are found in the cortex, occipital lobe, and thalamic regions [43]. In addition, the alpha waves play an important role in networking between neurons. It was reported that the alpha frequency is highly associated with cognitive performance [44]. In particular, the increases in alpha wave activity have also been correlated with an enhanced perception of calmness. Overall the alpha waves are connected with mental coordination, calmness, alertness, integration and learning states of the brain [45,46].

#### 4.1.4. Beta Waves

The frequency range of beta waves is 13–30 Hz and normally occurs during a heightened state of awareness. They are a fast wave activity and occur when we are alert, attentive and engaged in problem solving, decision making, and focused mental activity [47]. The beta waves are further divided into three bands such as low beta, mid beta and high beta. The beta waves occur once a task is being completed, and throughout active concentration. The activation of beta waves is mainly associated with benefits in academic performance and these waves significantly increase one’s cognitive skills. Further, the beta waves have also been reported to affect mental conditions. In general, beta wave activity decreases during the drowsiness state and increases during highly alert [48].

#### 4.1.5. Gamma Waves

Gamma waves usually occur at the frequency of >30 Hz and these brain waves are mainly involved in conscious attention with establishing neuronal circuitry. These are the fastest brain waves and correlate with simultaneous processing of information from different brain areas. They are usually found during working-memory matching and expanded consciousness, spiritual emergence and also during hypnotic states [49,50].

### 4.2. Brain Lobes (Regions) and Their Functions

The brain regions are categorized into frontal, temporal, parietal and occipital regions and each region has specific functions. However, many activities require coordination of multiple areas in both hemispheres.

#### 4.2.1. Frontal Region

The frontal region is located just beneath our forehead and it consists of all cortical areas anterior to the central sulcus. This region consists of a number of different functional areas such as the primary motor area, the premotor area and the prefrontal cortex [51]. The prefrontal region is the most important functional zone of frontal region. It regulates the physiological constructions of memory, perception and intricate action, and diverse cognitive processes. This region mediates a variety of higher cortical functions essential for planning, language, social interactions, and having a general executive oversight of other brain regions. Collectively, the frontal region is associated with a number of components including reasoning, planning, problem solving, parts of speech, intellect, behavior, attention, movements, sense of smell and personality [52,53].

#### 4.2.2. Parietal Region

The parietal region is situated between the frontal and occipital regions. This region contains the postcentral gyrus, superior parietal lobe, parietal operculum, supramarginal gyrus, and angular gyrus. This region is also divided into two functional areas: an anterior zone (somatosensory cortex) and a posterior zone (posterior parietal cortex). The parietal region plays important roles in integrating the sensory information from various parts of our body, understanding spatial orientation, recognition and perception of stimuli. The somatosensory cortex is essential for processing touch sensations and, especially helps to discriminate between sensations such as temperature and pain [54,55].

#### 4.2.3. Temporal Region

The temporal region is located near the ears and is mainly associated with auditory information, memory, emotion, conceptual understanding and in the perception of spoken and written language [51]. It was reported that the right hemisphere of this region is related to creative processing, and the left hemisphere is related to logical processing [56,57].

#### 4.2.4. Occipital Region

The occipital region is the most posterior portion of the human cerebral cortex. This region is mainly associated with visual information processing (reception, orientation, motion, and color recognition) and communication with the cerebral cortex. The surface area of the human occipital region is about 12% of the total area of the neocortex of the brain [51,58].

### 4.3. Administration of Fragrances

The EEG measurement room is maintained at a constant room temperature and humidity. The administration methods of fragrances to study the EEG activity were varied among the authors. In general, the fragrance administration has been carried out by a known volume of fragrance (diluted or undiluted), was dropped on the filter paper or perfume’s test strip and then placed about 3–10 cm in front of the subject’s nose [16,59,60]. Fragrances were also presented to the subject by a funnel-shaped supplier fixed on the chest (15 cm under the nose) with a flow rate of 2000 mL oil/min [61]. In another study, three drops of essential oil were added on a sterile dental swab and placed in a pierced metal container (6 cm wide × 6 cm long) and placed about 15 cm above the infant’s head and out of view from the infant [62]. Iijima et al. [14] administered the fragrances to the subjects by using the sample chamber placed 5 cm in front of the nose. Briefly, 0.05 g of fragrances were spotted on the filter paper and placed inside an 80 mL sample chamber, and then odorless air was pumped into the chamber (flow rate at 1 L/min).

Recently, some authors followed the odorant delivery system using a constant flow-olfactometer (flow rate at 1.0 L/min). Air from the chamber was transmitted through additional stainless tubing to a modified mask immobilized at 15 cm from the nose of a subject [15]. A particular amount of fragrance oil was administered using an oxygen pump system through a plastic tube via respiratory masks (flow rate at 2 L/min) [12]. In another report, a known volume of diluted fragrance oil was added in a plastic bag and administered through a mask [63]. In a recent study, a nebulizer was used for the administration of fragrance oil. The subjects were instructed to inhale the fragrance from the nebulizer nodule with a distance of approximately 10 cm between their nose and the nebulizer nodule [64].

### 4.4. EEG Measurement

**Figure 3:**
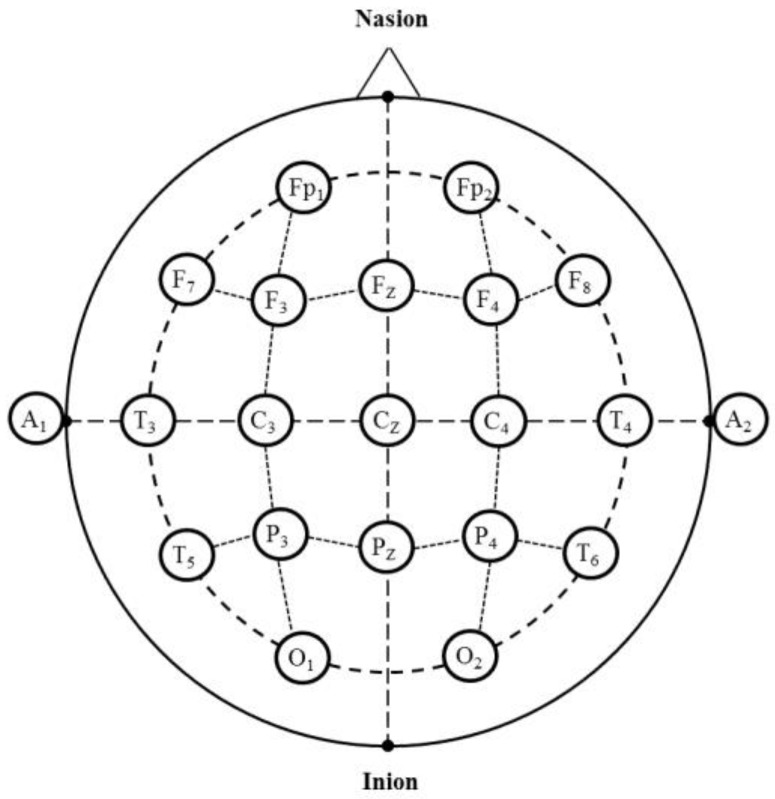
The electrode placement sites according to the international 10–20 system. Fp: frontopolar (Prefrontal); F: frontal; C: Central; T: temporal; P: parietal; O: occipital; A: auricular; z (zero): sagittal midline; odd numbers—left side, even numbers—right side.

For the EEG study, the International 10–20 system for electrode placement defines 21 standard scalp coordinates derived from four anatomical landmarks such as nasion, inion, and two preauricular points. The electrode placement regions are prefrontal or frontopolar (Fp), frontal (F), central (C), temporal (T), parietal (P), occipital (O) and auricular (A). In these sites, odd numbers are denoted to the left side, even numbers are denoted to the right side and zero (z) is referred to as the sagittal midline (Figure 3). The electrode placement sites on the scalp are expressed based on 10% or 20% of the distance between the nasion and inion, between the two pre-auricular points [35,65]. Most of the EEG recordings are based on simplified forms of signal data processing such as the Fast Fourier Transform. During the EEG measurement, the subjects are seated in a comfortable chair and are instructed to sit quietly, close their eyes and to breathe normally. The silver/silver chloride or gold electrodes are mainly used for the EEG study. In addition, the electro-caps made of an elastic spandex-type fabric are frequently used instead of individual electrodes. Depending on the purpose of the study, the EEG readings are recorded from different electrode sites according to the International 10–20 System [66].

Martin [59] used 28 electrodes attached to an elasticated cap placed on the scalp according to the International 10–20 System. The EEG signals were recorded for the frequency bands such as delta, theta, alpha 1, alpha 2, beta 1, beta 2 and beta 3. Total power was determined for the frequency range 0.5–30.0 Hz [13]. Masago et al. [61] recorded EEG readings from 12 scalp positions using multichannel biological amplifiers with a band-pass filtering between 0.1 and 32 Hz. Iijima et al. [14] employed the EEG recordings obtained from 21 shallow cup Ag electrodes placed on the scalp. The set of 31 electrodes were also used for the EEG study [12,16]. The electrodes included eight channels (Fp1, Fp2, F3, F4, T3, T4, P3 and P4), and a grounding electrode to the left earlobe, and a standard electrode to the right earlobe were frequently used in the EEG study [60,64].

The EEG recordings with eye blinks or motor artifacts were removed for each channel. All electrodes are referenced to the ipsilateral earlobe electrodes. The recording time for fragrance inhalation study is varied according to the authors (from few seconds to minutes). The standard bandwidth is 0.5–70 Hz with the 50 or 60 Hz notch filter. For standard EEG, the highest frequency is 70 Hz, and 200–256 Hz sampling is sufficient; the readings are stored in a computer by the analog to digital conversion [14,15,61]. The electrode gel is applied into each electrode to connect with the surface of the scalp in order to drop the electric resistance of the scalp below 5 kΩ. The mean power values are calculated as microvolts square (mV^2^), and the frequency bands such as absolute delta, theta, alpha, beta (low beta, mid beta and high beta) and gamma are recorded. The topographical mapping (t-mapping) of EEG brain waves was constructed by using software packages provided by the respective EEG instrument manufacturers. The t-maps may clearly illustrate differences which are difficult to understand in a table of EEG power spectra values. The statistical software packages are used for data analysis (Analysis of variance (ANOVA)/*t*-test) on EEG activity before and during the exposure of fragrances based on the EEG power spectrum values [33,36].

## 5. Effect of Inhalation of Fragrance on EEG Activity

Several studies have shown that the effects of various fragrances on mood, physiology and behavior are due to the fragrance’s direct and intrinsic ability to interact and affect the central nervous system. In addition, fragrances highly influence the various mental and physical conditions of human. The EEG recording is the simplest and precise technique to understand the effect of fragrances on brain function. In the EEG study, the subject is seated quietly and asked to inhale a fragrance of interest to the investigator. Subsequently, EEG data are gathered during this olfactory stimulation and later analyzed [4,5,34]. Table 2 shows the details of previous studies in relation to influences of aroma inhalation on human EEG activity.

Lorig [34,67] clearly reviewed the human EEG and odor response and discussed the association between olfaction and language. Van Toller [68] stated that the measurement of brain electrical activity using EEG recordings is now providing interesting new information on how odor signals are processed by the brain. Further, Martin [69] reviewed the therapeutic effects of odors on health-related behavior. Van Toller et al. [70] suggested that the EEG recordings from more anterior electrodes could be related to psychometric responses. Min et al. [71] found that the brains of professional perfume researchers respond to odors mainly in the frontal region, exhibiting the function of the orbitofrontal cortex due to the occupational requirement of these subjects to discriminate or identify odors. In another study, the combined form of Tai Chi/yoga significantly increased the relaxation state as well as tended toward an increase of EEG theta activity [72]. Herz [4] reviewed the scientific studies explaining olfactory effects on mood, physiology and behavior. The author clearly described the pharmacological and psychological hypotheses behind the previous reports. Freeman [73] stated that the sensory, motor, and hippocampal cortices interact intimately. Brain creates the contextual richness of relevant knowledge and expresses remembrances in spatial patterns of amplitude modification of beta and gamma waves.

Aromatic hydrocarbons are the most widely used solvents in the industries that cause central nervous system symptoms in exposed workers. Lorig et al. [74] conducted the effect of low concentration odor of galaxolide on central nervous system activity even when undetected. Significant differences were observed in alpha activity between the undetected odor and no odor control conditions. Further, the authors suggested that odors may be distracting or produce divided attention even when undetected. Seppalainen et al. [75] reported that during the early phase exposure of m-xylene increased the dominant alpha frequency and alpha percentage. Brauchli et al. [76] investigated the effect of phenylethyl alcohol (pleasant) and valeric acid (unpleasant) on EEG activity and suggested that smelling an unpleasant odor leads to a cortical deactivation by increasing alpha 2 power.

In the EEG study, the activation of alpha wave is the most important parameter and mainly influenced on the positive/negative psychological changes during the exposure of fragrant molecules. Masumoto et al. [77] suggested that the increasing trend of alpha wave after gum-chewing exhibited arousal psychosomatic responses. The lavender oil inhalation significantly decreased alpha 1 (8–10 Hz) activity at parietal and posterior temporal regions. Significant changes of alpha 1 were also observed after the inhalation of eugenol or chamomile. These data revealed that the decrease of alpha 1 activity is highly correlated with comfortable state of subjects [61]. Konagai et al. [78] investigated the effect of the aroma of soybeans heated to various temperatures in order to understand the relationship of the amino-carbonyl reaction products upon EEG. These results suggest that the aroma products from amino-carbonyl reaction increase alpha wave activity. Iijima et al. [79] found that the slow alpha (8–10 Hz) and theta activities significantly increased in the occipital region during the exposure to neroli oil and grapefruit oil when compared with activities before exposure and suggested that these oils reduce the cortical deactivation, or promote a relaxed state. In another study, it was reported that the odor of incense may enhance the cortical activities and the function of inhibitory processing of motor response by significantly increasing the fast alpha activity in bilateral posterior regions [14]. In general, the higher alpha wave activity is highly correlated with the reduced level of stress state.

In regards to theta wave activity, the changes in theta reflect modifications in attention or cognitive load, with a reduction in theta indicating a reduced level of attention. The effect olfactory stimulation of synthetic (chocolate, spearmint, almond, strawberry, vegetable, garlic, onion and cumin) and real food (chocolate, baked beans and rotting pork) odors on human central nervous system activity was investigated. Chocolate and spearmint odors significantly reduced the theta activity when compared with no-odor control [59]. Klemm et al. [80] reported that the odors of birch tar, jasmine, lavender and lemon significantly increased the theta activity. In addition, Schulz et al. [13] investigated the acute sedative effects of eight different plant extracts such as *Valeriana officinalis*, *Lavandula off.*, *Passiflora incarnata*, *Piper methysticum*, *Melissa off*., *Eschscbolzia californica*, *Hypericum perforatum* and *Ginkgo biloba*. Among the different plant extracts, valerian extract significantly increased delta and theta activities and also decreased beta activity. Boha et al. [81] studied the task-related EEG changes during the performance of a mental arithmetic task, as influenced by low alcohol dosages and reported that task-related significant theta power increase was observed particularly in the frontal area. Kawakami et al. [82] investigated the influence of fragrances with sedative effects (lemon, lavender and sandalwood) and awakening effects (jasmine, ylang-ylang, rose and peppermint) on visual display terminal task (VDT) activities. The results revealed that fragrances affected subject’s concentration on work and mentally stabilized them when compared with no fragrance control. Matsubara et al. [15] found that the essential oil of the Siberian fir tree, *Abies sibirica* (Pinaceae) increased theta activity after the VDT. These results showed that the essential oil of *A. sibirica* significantly reduced arousal levels after the VDT task.

Yagyu et al. [83] investigated brain electric field signatures of subjective feelings after chewing regular gum or gum base without flavor. Pre-post changes of source locations for the alpha 2 band and beta 2 band and of Global Field Power for delta to theta, alpha 2 and beta 1 decreased due to chewing regular gum and increased due to chewing gum. Subjective feeling changed to more positive values after regular gum when compared with gum base. Further, the author suggested that the chewing gum with and without taste or smell activates different brain neuronal populations. Further, Morinushi et al. [84] evaluated the effect of a chewing gum with and without flavor on the EEG activity. Chewing the standard gum base increased the alpha wave and decreased the beta wave. On the other hand, alpha and beta wave activities significantly increased when chewing the flavored standard gum as well as inhaling the flavored aromatic oil. In addition, significant change in the ratio of theta wave in the frontal area was observed when chewing the flavored standard gum. The results suggested that the flavor as well as chewing could enhance the concentration with a harmonious high arousal state of the brain function.

The isomers of aromatic compounds possess different fragrance qualities and intensities for humans. For example, in the case of enantiomers of 3-methylthiobutanal, one has a specific aroma and another one is odorless. Because of fragrance variations among the isomeric aroma components, their medicinal and biological properties also significantly varied [85,86,87]. The sedative properties of linalool were investigated using the optically active linalools ((*R*)-(−)-, (*S*)-(+)- and (*RS*)-(±)-forms). After hearing environmental sound, (*RS*)-(±)-linalool significantly decreased beta wave activity after work when compared with the before work and the same activity was also observed for (*R*)-(−)-linalool. However, the feature was just the reverse in the case of (*S*)-(+)-linalool [88]. In another study, Sugawara et al. [89] investigated the effects of inhalation of optically active linalools on humans in order to determine their odor distinctiveness by chiral isomers ((*R*)-(−)-linalools and (*S*)-(+)-linalools). From the results, the authors concluded that enantiomeric stereospecificity of linalool induced different odor perception and responses with chiral and task dependence. Sowndhararajan et al. [17] studied the olfactory stimulation of isomeric aroma components, (+)-limonene and terpinolene on EEG activity. According to gender variation, women responded well to both the compounds by a significant increase of absolute fast alpha activity. Further, the isomers exhibit diverse states of brain function as they affect different sites of the brain.

The EEG recordings were also used to investigate the influence of listening to soft music with/without inhaling *Citrus bergamia* aroma on the autonomic nervous system activity. The negative change of the ratio of low frequency to high frequency was observed in the music group, the aroma group, and the combined groups but not the control group. Listening to soft music and inhaling *C. bergamia* essential oil increased the relaxation state of brain [90]. Owen and Patterson [91] used the EEG recordings to investigate differences in hemispheric activation associated with different hedonic responses to a low concentration of a single compound (damascenone: fruity, berry smell). In their study, the results revealed that a non-significant trend for left frontal differences in EEG were due to different liking responses to damascenone, and these changes suggested quantification of the neurophysiological effects associated with odor liking. Watanuki and Kim [92] found that the beta wave activity increased in the left frontal brain region due to a pleasant odor. Patterson et al. [93] examined the relationship between odor responses of consumers to different flavor components (*para*-cresol 4-methylphenol, 2-heptanone, methional, 3-methylthiopropionaldehyde and dimethyltrisulphide). The authors suggested an ability of sensory ratings and brain recording techniques to reveal differences in responses associated with variations in background and experience.

In the psychophysiological properties of aroma, lavender is the most studied plant. The four important *Lavandula* species are *L. angustifolia*, *L. stoechas*, *L. latifolia*, and *L. intermedia*. The different lavenders have same ethnobotanical properties and major chemical constituents (geraniol, linalool, linalyl acetate, β-caryophyllene, β-ocimene, terpinen-4-ol and camphor) [94,95]. Lavender is mainly employed in aromatherapy treatments including inhalation, aromatherapy massage, dripping oil and bathing. Previous studies suggest that lavender has anxiolytic, mood stabilizer, sedative, analgesic and other neuroprotective properties [96]. Diego et al. [9] assessed the influence of lavender and rosemary on EEG activity, alertness, and mood. The authors suggested that the lavender group increased drowsiness by increasing beta power and the rosemary group increased alertness by decreasing frontal alpha and beta power. Further, lavender and rosemary fragrance may induce left frontal EEG shifting in adults and infants who show greater baselines relative to EEG activation in the right frontal region [97]. Fernandez et al. [62] investigated the effect of exposure of lavender or rosemary on EEG activity (based on EEG asymmetry) and suggested that infants of depressed and non-depressed mothers respond differently to odors. In addition, the effect of *L. angustifolia* aroma on the brain electrical activity in female adults with sleep disorders was investigated by Jung and Choi [98]. Results showed that *L. angustifolia* aroma decreased alpha activity in the occipital and parietal regions, and increased the theta and beta activities in the frontal and occipital regions, respectively, in subjects with good sleep quality. On the other hand, *L. angustifolia* aroma increased the theta activity in the all cranial regions in subjects with poor sleep quality. These data suggested that *L. angustifolia* aroma may have beneficial effect for female adults with sleep disorders.

Further, the inhalation of lavender oil resulted in more active, fresher, and relaxed subjects than those inhaling base oil. Lavender oil increased the theta and alpha wave activities when compared with base oil. The topographic map showed obviously more scattering power particularly in bilateral temporal and central area for alpha waves. These changes suggested the relaxing effect of inhaling lavender oil [12]. Recently, the effect of lavender (*L. angustifolia*) and bergamot (*Citrus bergamia*) essential oil inhalation on EEG recordings was studied by Lee [64]. The inhalation of essential oils significantly increased the absolute theta in the right prefrontal region. There were also significant differences in the relative fast and slow alpha after the inhalation of essential oil when compared with the control group. These EEG changes revealed that both the physical and mental states became more stable and relaxed after the inhalation of essential oil. Further, a mixture of lavender and bergamot oil was more effective than lavender oil alone. These reports clearly suggest that lavender oil may be an effective medicine in the treatment of various psychophysiological disorders.

In other studies, the essential oil from the seeds of *Zizyphus jujuba* significantly decreased the theta wave and increased the relative fast alpha, relative gamma, and spectral edge frequency 50%. Especially the relative fast alpha wave increased significantly in the left, right prefrontal, and left frontal regions during the inhalation of *Z. jujuba* essential oil. These changes suggested that the *Z. jujuba* seed oil increases the attention and relaxation states of brain [99]. In another study, Cho et al. [100] determined the effect of fragrant chemicals of essential oil from the aerial parts of *Mentha arvensis* L. f. *piperascens* on EEG activity. The relatively fast alpha activity significantly increased during the inhalation of *M. arvensis*. On the other hand, the values of gamma and the spectral edge frequency 90% were significantly decreased. The authors suggested that these EEG changes were associated with the reduction of mental stress. Further, Cho et al. [101] evaluated the effect of supercritical carbon dioxide extract of *Magnolia kobus* flower buds on EEG changes. During the inhalation of *M. kobus* fragrance, a significant decrease of absolute alpha wave was observed in the left parietal region. The results reveal alterations in EEG activity to awaken and enhance the concentration states of brain. Sayowan et al. [102] reported that the inhalation of jasmine oil increased the beta wave activity in the anterior center as well as the left posterior regions. These changes were associated with the increase of positive emotions such as the feeling of well-being, or feeling active, fresh and romantic. Watanabe et al. [63] found that impaired higher-order olfactory processing in temporal lobe epilepsy patients may inhibit the effects of the ylang-ylang aroma on the P300. Yoto et al. [103] investigated the memory task performance and the central nervous activity after smelling two kinds (Koushun and Kouju) of pan-fired Japanese green tea to examine their physical and psychological effects. The results showed that the odor of Kouju may induce a positive emotion. It may also affect the beta 1 activity at right frontal region and improve memory task performance.

Skoric et al. [16] examined human central nervous system response to the odors of lemon, peppermint, and vanilla. The theta wave activity showed significant difference due to the inhalation of fragrances and suggested that olfactory stimuli can affect the frequency characteristics of the electrical activity of the brain. Iannilli et al. [104] studied the electrophysiological response to food (strawberry) and non-food-related (lily of the valley) odors in healthy volunteers. The results showed specific scalp potential maps for the two conditions. The source of the map in the food condition seemed to be associated with the processing of rewards, whereas the specific map in the non-food condition reflects odor characteristics excluding the reward. Sowndhararajan et al. [60] investigated the effect of essential oil inhalation of *Inula helenium* root on human EEG activity. The absolute theta (all the regions except T3), beta (Fp1) and mid beta (P4) and relative theta (Fp1, Fp2, F3 and F4) wave activities significantly decreased during the essential oil inhalation compared to before inhalation. The changes in EEG activities due to the essential oil inhalation of *I. helenium* root may increase the alertness state of brain. 

The gender variation also plays an important role in the EEG recordings. Some of the previous studies suggested that the brains of male and female humans are differentially lateralized in relation to cognitive function. In addition, the EEG activity of resting males and females were different in the excitability dynamics of their cortical networks, and also gender differences were found in the stimulus and non-stimulus conditions [105,106]. Corsi-Cabrera et al. [107] studied the gender differences in the EEG during cognitive activity during rest and during solution of three series of tasks—analytic, spatial and mixed—demanding both kinds of processing. The results revealed that men showed significantly higher relative beta activity when compared to women, while women showed significantly higher relative alpha activity than men. Further, the gender variations are noticeable in event-related oscillations during simple visual stimulation [108]. Recently, Sowndhararajan et al. [17] reported that absolute and relative beta activities changed significantly more in men than women during the inhalation of isomeric components, (+)-limonene and terpinolene. In addition, the absolute fast alpha activity increased significantly more in women than men during the inhalation of these isomers. The previous reports clearly revealed that the variation in the EEG studies can be attributed to differences in EEG recording techniques and conditions, gender, as well as in the type and quality of fragrances administered.

## 6. Effect of Inhalation of Fragrance on Psychophysiological Activity

The positive effect of fragrances is mainly related to human behavior. The findings of the previously reported studies suggest that the olfactory system plays a major role in central nervous system functions. Angelucci et al. [5] reviewed the physiological effect of olfactory stimuli in humans. Hur et al. [109] also reviewed the evidence for the effectiveness of aromatherapy in the treatment of high blood pressure. In the present review, previously published studies on the relationship of aroma inhalation and psychophysiological functions of human are presented in Table 3. Roudnitzky et al. [110] investigated the interactions between texture and olfactory sensations, using a psychophysical and an electrophysiological approach. A butter aroma was presented either orthonasally or retronasally after oral processing and before swallowing the oral stimulus or in the absence of an oral stimulus. The authors suggested that the perceptual interactions occurred between food texture and odor, with cross-modal interactions being found for both orthonasal and retronasal odor administration. Further, these interactions between texture and odor occur at both primary-sensory and cognitive evaluative levels of stimulus processing. Hiessl and Skrandies [111] investigated the effect of food words such as odor, taste, vision or somatosensory texture and reported that the semantic dimensions influence neuronal processing of words in relation to multisensory perception. Sugawara et al. [112] clearly described the relationship between mood change, odor and its physiological effects in relation to verbal and non-verbal changes in humans induced by inhaling essential oils and individual components (linalool and its enantiomers).

Morris et al. [113] studied the anxiolytic effects of inhalation of geranium and rosemary. Sugawara et al. [114] reported the perceptional change of fragrance of essential oils such as ylang-ylang, orange, geranium, cypress, bergamot, spearmint and juniper in relation to type of work (mental work, physical work and hearing environmental sounds). The data confirmed that essential oil inhalation affected a different subjective sensitivity of fragrance depending on the type of work. In their study, inhalation of cypress after physical work produced a much more favorable impression than before work. For mental work, inhalation of juniper appeared to create a favorable impression after work. Lehrner et al. [115] stated that orange odor reduced anxiety and increased positive mood and calmness in women. Nagai et al. [116] investigated the effects of inhaling aromas (rose, jasmine and lavender) of preference on physical exercise in college students. The results revealed that the inhalation of preferred aromas suppressed the muscle sympathetic vasoconstrictor activity. In regards to sympathetic activity of aroma, essential oils from pepper, estragon, fennel or grapefruit increased relative sympathetic activity when compared with an odorless solvent (triethyl citrate). On the other hand, essential oils of rose or patchouli decreased relative sympathetic activity by 40% [117]. In addition to essential oil aromas, the individual odor components also affect the autonomic nervous system responses [118]. In general, human behaviors are closely linked to attention processes, which range from sleep to wakefulness. The aroma of essential oils such as peppermint, jasmine, ylang-ylang and individual essential oil components (1,8-cineole and menthol) significantly influenced basic forms of attention behavior [119]. Heuberger et al. [120] studied the influence of enantiomers of limonene and carvone (chiral fragrances) on the human autonomic nervous system and on self-evaluation. The author found that prolonged inhalation of fragrances affects autonomic nervous system parameters and states of brain. Further, the chirality of odor components appears to be a major factor in relation to the biological activity of fragrances.

Cavanagh and Wilkinson [121] reviewed the effect of lavender oils on psychophysiological properties. The essential oils obtained from various species of *Lavandula* have been used in cosmetics and pharmaceutical industries for centuries. Among the various species, the *L. angustifolia*, *L. latifolia*, *L. stoechas* and *L. x intermedia* are the most commonly used plants. Motomura et al. [122] also suggested that lavender aroma significantly reduced the stress and enhanced the arousal states of brain. In patients with severe dementia, an aroma stream with lavender oil shows modest efficacy in the treatment of agitated behavior [123]. Moss et al. [124] evaluated the olfactory effect of lavender and rosemary essential oils on cognitive performance and mood. In their study, the members of both the control and lavender groups were considerably less alert than the rosemary condition. It was reported that rosemary and lavender scents were associated with lower mean ratings on the fatigue-inertia subscale [125].

Campenni et al. [126] studied the effects of lavender (relaxing odor) and neroli (stimulating odor) on mood. Relaxing odor decreased heart rate and skin conductance, whereas stimulating odor produced the reverse effects under equivalent conditions. Gedney et al. [127] reported that lavender reduced the pain intensity and pain unpleasantness after treatment. Further, lavender serves as a mild sedative and is used for enhancing deep sleep in young men and women [128]. Influence of orange and lavender essential oils on anxiety, mood, alertness and calmness in dental patients was investigated by Lehrner et al. [129]. The results revealed that, compared to control conditions, both odors of orange and lavender reduced anxiety and improved mood in patients waiting for dental treatment. In another study, the impact of lavender-scented bath oil on mothers and their infants was reported. During the bath with lavender-scented oil, the mothers touched their infants for a longer amount of time, were more relaxed and smiled more. In addition, their infants cried less and spent more time in deep sleep after the bath. These behavioral data suggest that lavender increased relaxation state of the mothers and their infants [130,131]. Kim et al. [132] reported that lavender significantly decreased stress levels and the bispectral index values as well as the pain intensity of needle insertion. Sakamoto et al. [133] investigated whether exposure to aromas (jasmine and lavender) during recess periods affects work performance. In their study, lavender significantly increased concentration levels, but jasmine did not produce such an effect. In addition, Rho et al. [134] reported that aromatherapy (lavender, rosemary, lemon and chamomile) massage produced beneficial effects on anxiety and self-esteem in Korean elderly women.

In the athletic task performance, the peppermint odor significantly increased running speed, hand grip strength, and number of push-ups, but had no effect on skill-related tasks [135]. Raudenbush et al. [136] studied the effects of odor (peppermint oil, jasmine oil and dimethyl sulfide) administration on objective and subjective measures of physical performance of athletes. From the results, peppermint odor exhibited more slow-wave sleep and more total sleep and it also produced gender-differentiated responses [137]. In addition, the effect of peppermint oil on exercise performance in young male college students was reported by [138]. Ho and Spence [139] found a significant performance improvement in the presence of peppermint odor. Norrish and Dwyer [140] reported that the presence of peppermint oil controlled the increase in sleepiness during 11 min spent in a darkened room when compared with a no-odor condition. Moss et al. [141] provided the evidence for the impact of the aromas of plant essential oils (ylang-ylang aroma, peppermint aroma) on aspects of cognition and mood. Peppermint significantly enhanced memory and alertness. On the other hand, ylang-ylang lengthened processing speed and increased calmness.

Hongratanaworakit and Buchbauer [142] studied the effects of transdermal absorption of ylang-ylang oil on physiological parameters and self-evaluation in human. The ylang-ylang oil significantly decreased the blood pressure and increased the skin temperature. Further, subjects in the ylang-ylang oil group rated themselves calmer and more relaxed when compared to subjects in the control group. Moss et al. [143] examined the effect of the aroma of the essential oil of Roman chamomile (*Chamaemelum nobile*) on mood and cognition in human. The authors stated that the subjective alertness was associated with the sedative effect of the aroma and subjective calmness was associated with both the aroma’s sedative effect and stimulated arousal expectancy. Kohler et al. [144] stated that caffeine effectively improved speed and accuracy on cognitive tasks and increased alertness when compared with chewing.

Hummel and Heilmann [145] investigated the perception of odor (chocolate and lavender) intensity through ortho- and retronasal presentation. The findings of their study suggested that the response was larger when an odor unrelated to food was presented in an unusual site (retronasally) compared with presented in an orthonasal site. The authors stated that the route of odor presentation has direct associations with the enjoyment of foods and drinks. Yamagishi et al. [146] investigated the influences of heliotropin on nighttime sleep and suggested that this aromatic compound effectively improves sleep. The potential pharmacological relationships between absorbed 1,8-cineole followed by rosemary aroma exposure and mental behavior were studied by Moss and Oliver [147]. The data revealed that different neurochemical pathways were responsible for their action on cognition and subjective state. Jun et al. [148] reported that the inhalation of eucalyptus oil effectively decreased patient’s pain and blood pressure after total knee replacement surgery.

Liu et al. [149] used natural bergamot essential oil extracted from plants and synthesized a chemical essential oil to study their aromatherapy effect in relieving work-related stress. The results showed that the natural bergamot essential oil relieved work-related stress of teachers with various workloads. However, the treatment showed a weak effect on young teachers with a heavy workload. Iannilli et al. [104] investigated the electrophysiological response to food- and non-food-related odors in healthy volunteers and the analyses revealed the specific scalp potential maps for the two conditions. Sugawara et al. [150] elucidated the psychophysiological effect of inhaling 12 different essential oils and suggested that essential oils may have versatile psychophysiological properties.

From the literature of previous studies, lavender, peppermint, rosemary, jasmine, ylang-ylang, lemon, geranium, chamomile and spearmint are the most studied aromatic plants. These plants have been used in the aromatherapy for the treatment of various psychological and physiological disorders [1].

## 7. Conclusions

Based on the previous studies, it can be concluded that fragrances directly and/or indirectly affect the psychological and physiological conditions of humans. In addition, the electroencephalograph studies clearly revealed that fragrances significantly modulate the activities of different brain waves and are responsible for various states of the brain. Further, a number of studies have scientifically supported the beneficial use of various aromatic plants in aromatherapy. However, this study in relation to fragrance stimulation on EEG activity has some limitations. The concentration of the fragrances also plays a major role in EEG activity, because a higher concentration provides a higher fragrance density. Hence, results may differ when using different concentrations of the fragrance. Moreover, the EEG recording time is a very important factor in attaining constant EEG readings from various laboratories. Therefore, it is still unknown whether the fragrances will show the same effect for a longer duration of EEG recordings with different concentrations and more participants. In light of these limitations, standardizing and developing a common standard operating procedure for the effect of fragrances on EEG activity (such as recording time, administration method, concentration of fragrance, number of electrode sites and placebo) is necessary. Only then will we be able to understand the exact action of fragrances on human brain function in relation to EEG brain wave changes.

## Figures and Tables

**Table 1:** Some of the important essential oil-bearing plants and their major components.

Plants Name	Botanical Name	Major Components
Bergamot	*Citrus bergamia*	limonene, linalool, linalyl acetate
Caraway	*Carum carvi*	carvone, limonene
Chamomile	*Matricaria chamomilla*	α-bisabolol, bisabolol oxide B, (*E*)-β-farnesene, α-bisabolone oxide
Cinnamon	*Cinnamomum zeylanicum*	cinnamaldehyde, cinnamyl acetate
Cornmint	*Mentha arvensis*	menthol, menthone, isomenthone, menthyl acetate
Eucalyptus	*Eucalyptus* sps.	1,8-cineole (eucalyptol), limonene, aromadendrene
Geranium	*Pelargonium graveolens*	citronellol, geraniol, citronellyl formate, linalool
Jasmine	*Jasminum* sps.	benzyl alcohol, linalool, benzyl acetate, jasmone, geraniol
Juniper	*Juniperus* sps.	bornyl acetate, sabinene, α-pinene, limonene
Lavender	*Lavandula* *angustifolia*	geraniol, linalool, linalyl acetate, β-caryophyllene
Lemon	*Citrus limon*	limonene, β-pinene, γ-terpinene, p-cymene
Lemongrass	*Cymbopogon citratus*	citral (geranial), neral, myrcene
Oregano	*Origanum vulgare*	carvacrol, thymol, cymene
Palmarosa	*Cymbopogon martinii*	geraniol, geranyl acetate, linalool
Peppermint	*Mentha piperita*	menthol, menthone, 1,8-cineole, menthofuran
Pine	*Pinus* sps.	α-humulene, caryophyllene, β-pinene, β-cadinene
Rose	*Rosa damascena*	citronellol, geraniol, β-pinene, rose oxide
Rosemary	*Rosmarinus officinalis*	camphor, 1,8-cineole, α-pinene, borneol, camphene, β-phellandrene
Sandalwood	*Santalum album*	α-santalol, β-santalol, β-curcumen-12-ol
Spearmint	*Mentha spicata*	carvone, 1,8-cineole, limonene
Sweet basil	*Ocimum basilicum*	linalool, α-cadinol, α-bergamotene, γ-cadinene
Thyme	*Thymus vulgaris*	thymol, carvacrol, terpinene, cymene
Ylang-ylang	*Cananga odorata*	geranyl acetate, benzyl benzoate, eugenol, germacrene-d, geraniol

**Table 2:** Effect of inhalation of aroma on electroencephalograph (EEG) activity.

S. No.	Odorant Materials	EEG Wave Changes	Brain Functions	Reference
1.	Galaxolide	Alpha decreased.	Odors produce divided attention even when undetected.	[74]
2.	*m*-Xylene	Alpha increased.	Stimulating and excitatory effects.	[75]
3.	Birch tar, galbanum, heliotropine, jasmine, lavender, lemon and peppermint	Increased theta for birch tar, jasmine, lavender and lemon.	Subjects differed in their subjective responses to the odors.	[80]
4.	5-α-Androstan-3-one, bangalol, white sapphire, indole, linalyl acetate, eucalyptus oil and ammonia.	Alpha increased.	From more anterior electrodes—related to psychometric responses.	[70]
5.	Phenylethyl alcohol and valeric acid	Valeric acid—alpha 2 increased.	Unpleasant odor leads to a cortical deactivation.	[76]
6.	Lavender and rosemary	Lavender—beta increased. Rosemary—frontal alpha and beta decreased.	Lavender—increased drowsiness. Rosemary—increased alertness.	[9]
7.	Synthetic odors—almond, chocolate, spearmint, strawberry, vegetable, garlic, onion and cumin Odors of real foods—chocolate, baked beans and rotting pork	Chocolate odor—less theta activity.	Reduced level of attention.	[59]
8.	Chewing of marketed gum	Alpha power increased.	Arousal psychosomatic responses.	[77]
9.	*Valeriana off*, *Lavandula off, Passiflora incarnata*, *Piper methysticum*, *Melissa off, Eschscbolzia californica*, *Hypericum perforatum* and *Ginkgo biloba*	Valerian extract—delta and theta activity increased and beta activity decreased.	Self-rated tiredness increased under some of the plant extracts.	[13]
10.	(*R*)-(−)-, (*S*)-(+)- and (*RS*)-(±)-forms of linalools	*(RS)*-(±)-linalool—greater decrease of the beta wave after work than before work.	(*RS*)-(±)-linalool and (*R*)-(−)-linalool -favorable impression. (*S*)-(+)-linalool—unfavorable impression.	[88]
11.	Chewing regular gum or gum base without flavor	Alpha-2 and beta-2 increased for regular gum and decreased for gum base.	Activates different brain neuronal populations.	[83]
12.	Sedative effects—lemon, lavender and sandalwood Awakening effects—jasmine, ylang-ylang, rose and peppermint	Awakening fragrances—decreased alpha and beta activities.	Sedative fragrances—improvement in productivity. Awakening fragrances—effect in mitigating the workload.	[82]
13.	Lavender, chamomile, sandalwood and eugenol	Alpha 1 decreased at parietal and posterior temporal regions.	Subjects felt comfortable.	[61]
14.	Chewing gum with and without flavor and flavored aromatic oil	Chewing gum with flavor and inhale aromatic oil increase alpha and beta waves.	Induce concentration with a harmonious high arousal state in brain function.	[84]
15.	Enantiomers of linalools	(*R*)-(−)-linalool—beta decreased after hearing environmental sound. Mental work—beta increased.	Odor perception and responses—chiral dependence and also with task dependence.	[89]
16.	Aroma of soybeans heated to various temperatures	Alpha wave increased—heated after immersion in fructose–glycine solution.	Amino-carbonyl reaction aroma products increase brain alpha waves.	[78]
17.	β-Damascenone	Non-significant trend for left frontal differences in EEG associated with different liking responses.	Left frontal response associated with liking an odor.	[91]
18.	Lavender and rosemary aromas	Induce left frontal EEG shifting in adults and infants with greater baselines than right frontal EEG activation.	Associated with greater approach behavior and less depressed affect.	[97]
19.	General workers, perfume salespersons and professional perfume researchers	Professional perfume researchers respond to odors mainly in the frontal region.	Functional coupling for people—occupationally exposed to odors may be related to psychological preference.	[71]
20.	Lavender and rosemary	Increased relative left frontal EEG asymmetry.	Infants of depressed and non-depressed mothers respond differently to odors.	[62]
21.	Para-cresol 4-methylphenol, 2-heptanone, methional 3-methylthiopropionaldehyde and dimethyltrisulphide.	Theta wave activation in frontal region between the different populations.	Cultural differences in odor responsiveness.	[93]
22.	Pleasant odor	Beta wave increased in the left frontal region.	Enhancement of left frontal brain region by a pleasant odor.	[92]
23.	Neroli and grapefruit oils	Slow alpha (8–10 Hz) and theta activities increased in the occipital region.	Reduce the cortical deactivation or promote a relaxed state.	[79]
24.	Low-dose alcohol	Theta power decreased in both hemispheres in the high-dose condition.	Corresponding to working memory demand.	[81]
25.	Odor of incense and rose oil	Fast alpha activity increased in bilateral posterior regions during incense exposure.	Cortical and function of inhibitory processing of motor response.	[14]
26.	*Citrus bergamia* oil	Negative percentage changes of the ratio of low to high frequency in the music, aroma and combined groups than control group.	Listening to soft music and inhaling *Citrus bergamia* essential oil—effective method of relaxation.	[90]
27.	*Abies sibirica* essential oil	Increased theta activity after the visual display terminal task.	Prevention of visual display terminal—mental health disturbance.	[15]
28.	*Lavandula angustifolia*	Good sleep quality—occipital and parietal alpha decreased, frontal theta and occipital beta increased. Poor sleep quality—theta increased in the all cranial regions.	Beneficial effect for female adults with sleep disorder.	[98]
29.	Lavender oil	Theta and alpha activities increased.	Relaxing effect of inhaling lavender oil.	[12]
30.	Essential oil of *Zizyphus jujuba* seeds	Fast alpha increased in the left prefrontal, right prefrontal and left frontal regions.	Increasing attention and relaxation.	[99]
31.	Essential oil of *Mentha arvensis* L. f. *piperascens* aerial parts	Relative fast alpha increased. Gamma and the spectral edge frequency 90% decreased.	Reducing mental stress.	[100]
32.	Jasmine oil	Beta wave increased in the anterior center and left posterior regions.	Increased—feeling of well-being, active, fresh and romantic.	[102]
33.	Ylang–ylang essential oil	Prolonged the latencies of P300	Not affect information processing resources in patients with TLE.	[63]
34.	Essential odors—mint and lemon Commerical odors—criton-verbena, lize, melody and rozan	All odors affected the EEG waves in at least some subjects.	Essential odors stimulated more than commercial odors and women are more sensitive than men.	[35]
35.	Pan-fired Japanese green tea (Koushun and Kouju)	Kouju affect the beta 1 at right frontal region.	Improve memory task performance.	[103]
36.	*Magnolia kobus* flower	Absolute alpha decreased at left parietal region.	Awaken and increase the concentration states of brain.	[101]
37.	Strawberry aroma (food) and the odor of lily of the valley (non-food)	Specific scalp potential maps for the two conditions.	Food odor—associated with the processing of rewards. Non-food odor—reflects odor characteristics excluding the reward.	[104]
38.	Hyperbaric oxygen exposure	Fast delta decreased and alpha increased in the posterior regions.	Oxygen-toxicity diving-related problems.	[151]
39.	Lemon, peppermint, and vanilla	Theta showed statistically significant results between different odor conditions	Stimuli can affect the frequency characteristics of the electrical activity of the brain.	[16]
40.	Isomers of limonene and terpinolene	(+)-Limonene—relative high beta increased in the right temporal region. Terpinolene—relative mid beta decreased and relative fast alpha increased in the right prefrontal region.	Terpinolene—reducing the tension and increasing the relaxation and stabilization states of brain function.	[17]
41.	Essential oil of *Inula helenium* root	Theta (in all the regions except T3), beta (Fp1) and mid beta (P4) and relative theta (Fp1, Fp2, F3 and F4) decreased.	Enhance the alertness state of brain.	[60]
42.	Lavender and bergamot	The absolute theta increased at the right prefrontal region Significant differences in the relative fast and slow alpha.	Both physical and mental states became more stable and relaxed.	[64]

**Table 3:** Effect of inhalation of aroma on psychophysiological functions of human.

S. No.	Odorant Materials	Psychophysiological Changes	Reference
1.	Rosemary and geranium oil	Geranium oil inhalation—both state and trait scores were significantly lowered.	[113]
2.	Ylang-ylang, orange, geranium, cypress, bergamot, spearmint, and juniper	Cypress produced favorable impression after physical work and juniper produced favorable impression after mental work.	[114]
3.	Orange	Relaxant effect—lower level of state anxiety, a more positive mood, and a higher level of calmness.	[115]
4.	Rose, jasmine and lavender	Inhalation of favorite odors suppresses the muscle sympathetic vasoconstrictor activity and attenuates the blood pressure increase by affecting the central nervous system higher than the midbrain.	[116]
5.	Enantiomers of limonene and carvone	Carvone—subjective restlessness. Prolonged inhalation of fragrances influences autonomic nervous system parameters as well as mental and emotional conditions.	[120]
6.	Peppermint, jasmine, ylang-ylang, 1,8-cineole and menthol	Essentials oils or their components on basic forms of attention behavior are mainly psychological.	[119]
7.	Lavender	Lavender odorants were associated with reduced mental stress and increased arousal rate.	[122]
8.	Peppermint	Enhanced physical performance and generating more push-ups and running faster.	[135]
9.	Isovaleric acid, thiophenol, pyridine, l-menthol, isoamyl acetate, and 1,8-cineole	Autonomic variations in response to olfactory stimuli—along two main dimensions of pleasantness and arousal.	[118]
10.	Pepper oil, estragon oil, fennel oil or grapefruit oil, rose oil or patchouli oil	Fragrance inhalation of rose oil or patchouli oil caused a 40% decrease in relative sympathetic activity.	[117]
11.	Lavender oil	Treatment of agitated behavior in patients with severe dementia.	[123]
12.	Peppermint oil, jasmine oil and dimethyl sulfide	Peppermint odor reduced received work load and effort and increased self-evaluated physical performance and energy.	[136]
13.	Lavender and rosemary oils	Lavender produced a significant decrease in performance of working memory. Rosemary produced a significant enhancement of performance for overall quality of memory.	[124]
14.	Rosemary and lavender oils	Both rosemary and lavender scents were associated with lower mean ratings on the fatigue-inertia subscale, relative to the control group.	[125]
15.	Lavender and neroli	Relaxing odors decreased heart rate and skin conductance, with stimulating odors producing reverse effects under equivalent conditions.	[126]
16.	Lavender and rosemary oils	Alter affective appraisal of the experience and consequent retrospective evaluation of treatment-related pain.	[127]
17.	Lavender oil	Increased the percentage of deep or slow-wave sleep in men and women and decreased rapid-eye movement sleep.	[128]
18.	Peppermint oil	Reduced fatigue and improved mood and was rated as more pleasant, intense, stimulating, and elating than water.	[137]
19.	Synthetic peppermint odor	Performance improvement in the presence of peppermint odor when the response mapping was incompatible but not in the compatible condition.	[139]
20.	Jasmine tea, lavender, (*R*)-(−)-linalool and (*S*)-(+)-linalool	Jasmine tea, lavender and (*R*)-(−)-linalool increased positive mood state.	[152]
21.	Orange and lavender	Reduced anxiety and improved mood in patients waiting for dental treatment.	[129]
22.	Peppermint oil	Daytime sleepiness, peppermint oil can indeed reduce sleepiness.	[140]
23.	Lavender and Jasmine	During recesses—higher concentration levels for lavender group	[133]
24.	Ylang-ylang oil	More calm and more relaxed.	[142]
25.	Chewing and caffeine	Caffeine led to improved speed and accuracy on cognitive tasks and increased alertness when compared with chewing.	[141]
26.	Essential oil of Roman chamomile	Sedative effect	[143]
27.	Lavender, chamomile, rosemary, and lemon	Aromatherapy massage exerts positive effects on anxiety and self-esteem.	[134]
28.	With or without lavender-scented bath oil	Mothers—more relaxed, and smiled. Infants—cried less and spent more time in deep sleep.	[130]
29.	Lavender	Increased relaxation.	[131]
30.	Lavender and chocolate odors	Ortho- and retronasal odor presentation—route of odor presentation has direct implications for the enjoyment of foods and drinks.	[145]
31.	Ylang-ylang aroma, peppermint aroma	Peppermint enhanced memory whereas ylang-ylang impaired it, peppermint increased alertness and ylang-ylang decreased it.	[141]
32.	Aroma of heliotropin	Reduced sleepiness and improved refreshment at awakening for participants who suffered from insomniac symptoms.	[146]
33.	Lavender oil	Reduced the stress levels and the pain intensity of needle insertion.	[132]
34.	1,8-Cineole following exposure to rosemary aroma	Cognitive tasksare significantly related to concentration of absorbed 1,8-cineole following exposure to rosemary aroma.	[147]
35.	*Eucalyptus* oil	Pain and inflammatory responses after total knee replacement.	[148]
36.	Bergamot essential oil and synthetic oil	Relieved work-related stress of teachers with various workloads and had a weak effect on young teachers who had a heavy workload.	[149]
37.	Peppermint oil	Relaxation of bronchial smooth muscles, increased ventilation and brain oxygen concentration, and decreased blood lactate level.	[138]
38.	Basil, bergamot, cardamom, cinnamon, juniper, lemon, orange, plamarosa, peppermint, sandalwood, spearmint and ylang-ylang	Essential oils may have versatile psychophysiological potencies.	[150]
